# The Use of Positive Psychology in Studies with Healthcare Professionals: Scoping Review and Implications for Professional Support

**DOI:** 10.3390/ijerph23030296

**Published:** 2026-02-27

**Authors:** Wanderlei Abadio de Oliveira

**Affiliations:** Graduate Program in Psychology, School of Life Sciences, Pontifical Catholic University of Campinas, Avenida John Boyd Dunlop, Jardim Ipaussurama, Campinas 13060904, SP, Brazil; wanderleio@hotmail.com

**Keywords:** healthcare worker, health care professional, positive psychology, mental health, burnout, psychology, review

## Abstract

**Highlights:**

**Public health relevance—How does this work relate to a public health issue?**

- Work-related illness among healthcare professionals represents a critical public health issue, as it directly affects the quality, continuity, and safety of care provided to populations.
- Addressing this issue also requires the consideration of individual-level factors such as personal resources, coping strategies, and psychological strengths. By incorporating these dimensions into research and intervention design, it becomes possible to develop more effective and comprehensive approaches that integrate both systemic and individual determinants of occupational health.

**Public health significance—Why is this work of significance to public health?**

- This work is significant because it addresses the well-being of healthcare professionals as a key factor in the quality and continuity of care.
- By integrating psychological dimensions into the analysis of occupational stress, the study broadens the scope of public health approaches, supporting interventions that promote emotional well-being, prevent illness, and strengthen the workforce at both individual and systemic levels.

**Public health implications—What are the key implications or messages for practitioners, policy makers and/or researchers in public health?**

- There is a need to prioritize pre-service and in-service training models that proactively support healthcare workers’ well-being, rather than relying solely on reactive interventions aimed at treating stress after it has developed.
- Findings of the scoping review provide evidence-based insights for policy and practice, highlighting interventions that (a) improve emotional and psychological well-being, (b) strengthen personal resources through reflection, and (c) foster professional respect and a positive organizational climate.

**Abstract:**

Twenty-five years after the inception of Positive Psychology, its principles are still perceived to be underutilized in addressing working conditions or providing care to healthcare professionals. This article aims to synthesize evidence on the use of Positive Psychology in supporting healthcare workers. A detailed data search and analysis strategy was employed to identify scientific evidence that could answer the following guiding question: What empirical evidence is available regarding the effects of applying Positive Psychology principles or interventions to the mental health and well-being of healthcare professionals? Ten articles were selected and analyzed using the META-CORE model (Meta-level Conceptual, Operational, Reflective Evaluation). Based on the main findings of the ten reviewed studies, three themes were developed: (1) promotion of emotional and psychological well-being, gathering evidence that reflects subjective changes among healthcare professionals; (2) strengthening of personal resources and virtues through a process of self-perception and self-assessment within the work context; and (3) professional appreciation and a positive organizational climate. This scoping review contributes to strengthening the theoretical foundation of current attention to the situation of healthcare workers, aligning more clearly with the conceptual bases of Positive Psychology and its concern with mental health and the growing imperative to address contemporary challenges.

## 1. Introduction

The healthcare field is recognized as a demanding and multifaceted work environment, where professionals frequently face high physical, emotional, and organizational demands [1]. Psychosocial factors such as long working hours, intense emotional demands, high-risk settings, and insufficient institutional support contribute to increasing the psychological distress experienced by health workers [2,3]. Among these factors, work overload is one of the most frequently explored issues in scientific literature, encompassing high patient care demands, staff shortages, long shifts, emotional pressure related to clinical decision-making, interprofessional tensions, and limited resources [4]. Other barriers reported by healthcare professionals include lack of training, ineffective teamwork, insufficient institutional and governmental support, unclear responsibilities, and social inequalities, among others.

This context shows that working in healthcare impacts not only physical well-being but also has significant consequences on mental health, often leading to increased rates of mental disorders and, notably, burnout syndrome. This has been documented in the literature, although not always extensively or consistently across contexts. For instance, a cross-sectional study involving 959 Italian healthcare workers identified 328 suspected cases of anxiety (34.2%) and 334 cases of depression (34.8%), both unreported or undiagnosed [5]. Such experiences of suffering and extreme exhaustion, often resulting from chronic and excessive work-related stress, are identified as burnout syndrome [6]. This occupational phenomenon has been explored in the literature, although the scope and depth of investigation vary across studies and contexts. For example, in Uganda, burnout rates ranged from 16% to 86%, with an overall average of 57.4% [7]. In Brazil, occupational stress and burnout prevalence are also high [8]. Similar patterns are observed across Europe, where elevated burnout rates among healthcare professionals are consistently reported.

It is important to note that this scenario of illness is not necessarily related to the level of healthcare complexity but rather to the intensity of human interaction and caregiving responsibilities. In fact, inadequate training may exacerbate this condition by making caregiving tasks simultaneously complex and intense, reinforcing the need to consider how these dimensions interact in shaping occupational stress. In other words, health professionals at various levels of care, from primary care to intensive care units, are equally vulnerable to burnout and mental distress. Community health workers (CHWs), for instance, often have limited formal training and maintain strong ties with their local communities [9], making them especially susceptible to job-related stress and burnout syndrome [10]. On the opposite end of the healthcare system, a recent literature review reported burnout prevalence of 50% among intensive care unit nurses [11].

Health issues have direct implications for reduced work capacity [5]. On the other hand, personal characteristics must also be considered to understand how workplace demands and challenges interact with individual resources, either increasing vulnerability to illness or offering potential pathways to well-being and resilience. This perspective highlights that personal attributes may influence how an individual copes with workplace stress. From this standpoint, Positive Psychology was chosen as the theoretical framework for this study, as it offers conceptual and empirical tools to understand and promote individual strengths which are crucial in managing occupational challenges.

### 1.1. Positive Psychology in Healthcare

Positive psychology emerged as a critical response to the historically predominant focus of psychology on pathologies and negative emotional states. In 2000, the scientific journal American Psychologist published a Special Issue dedicated to this new approach, defining positive psychology as the study of positive emotions, character strengths, and institutions that promote human flourishing [12]. This publication marked the beginning of an academic movement that began to emphasize human potential and the factors that contribute to a meaningful and satisfying life. In terms of definition, it is a field of psychology devoted to the scientific study of the qualities and conditions that support healthy development and human well-being [13]. This area seeks to investigate positive emotions, such as gratitude and hope, personality traits like resilience and optimism, and social contexts that foster human flourishing [12,13].

In practice, positive psychology is applied, for instance, in mental health promotion programs for healthcare professionals, by encouraging the practice of gratitude or the recognition of personal strengths as a way to reduce occupational stress. It can also be seen in schools through activities that strengthen a sense of purpose and positive interpersonal relationships among students. This movement represented a significant advancement by expanding psychology’s scope beyond the treatment of psychological or mental health problems, also focusing on strengthening mental and emotional health, as well as on the more positive aspects of development [12,13,14]. By redirecting scientific attention to personal strengths, virtues, and well-being, positive psychology positions itself as a paradigmatic shift within the field of psychology [14].

Within this theoretical framework, character strengths emerge as a central principle. They are defined as pathways to the major human virtues, including positive personality traits that reflect identity and are capable of producing positive outcomes for both the individual and those around them [15]. In the classical model, these strengths are organized into six universal virtues: wisdom/knowledge (cognitive virtue), courage (emotional virtue), humanity (interpersonal virtue), justice (civic virtue), temperance (protective virtue), and transcendence (spiritual virtue) [16]. Another important concept is mindset, which refers to the underlying beliefs individuals hold about their abilities and influences how they perceive and respond to challenges. Research has already shown that character strengths are associated with life satisfaction, flourishing, subjective and psychological well-being, lower levels of stress, and reduced emotional suffering, among other benefits.

Concurrent to these aspects, it is worth noting that in adulthood, work represents one of the main activities in human development. It is understood as an expenditure of vital energy that occurs when professional demands are met in the workplace. It is important to emphasize that this energy directed toward professional activities is subject to the inherent depletion caused by the tension of demands and the fatigue resulting from the execution of work. Therefore, it is essential that this energy be regularly replenished to ensure the creation of a healthy work environment [17]. According to positive psychology, two underlying processes occur simultaneously in the work context: first, a motivational and energetic process that results in work engagement; second, a process of wear and stress that may lead to burnout [17]. The interaction between job demands and resources thus constitutes the central factor for a deeper understanding of work dynamics and their impact on workers.

### 1.2. The Present Study

Initially, this review was conceived to identify interventions based on the principles of positive psychology developed for or focused on the treatment of healthcare professionals. However, during preliminary database searches, no results were found. Given this, the study’s objective was revised to synthesize evidence on the use of positive psychology in the care of healthcare professionals. Based on the findings, this study also aims to reflect on implications for promoting the mental health of healthcare workers, considering the identified gap in intervention-based studies.

## 2. Methods

### 2.1. Design

The present study followed a scoping review design, an approach recommended for systematically and comprehensively mapping an emergent or heterogeneous body of literature on a given topic. This type of review employs an iterative and rigorous process that is particularly suitable for synthesizing the scope, nature, and gaps in the available knowledge [18]. The methodological design was conducted in accordance with the PRISMA-ScR (Preferred Reporting Items for Systematic Reviews and Meta-Analyses extension for Scoping Reviews) checklist, ensuring transparency, reproducibility, and standardization in the procedures of searching, screening, extracting, and synthesizing data [19]. The PRISMA checklist is included as a Supplementary Material. The review protocol was pre-registered and published on the Open Science Framework (OSF), under the DOI 10.17605/OSF.IO/6UCKE. The Rayyan platform was used to organize and conduct the screening and selection of studies [20]. Ethical approval was not required due to the nature of the study, which did not involve human or animal subjects. Nonetheless, all ethical principles were carefully observed, especially regarding citation, referencing, and appropriate acknowledgment of the authors consulted.

### 2.2. Research Question

The formulation of the guiding research question was informed by the PICO strategy, which is widely used in evidence-based research to structure questions in a clear, focused, and searchable format [21]. PICO is an acronym that refers to four essential components: Population or Problem (P), Intervention (I), Comparison (C), and Outcome (O). This structure is particularly useful in studies that aim to measure the effects of specific interventions, as it enables researchers to precisely define the central elements that should be considered in the literature searches and in the analysis of findings. Applying this framework to the objective of the present review, the following elements were defined: P = healthcare professionals; I = principles and interventions based on Positive Psychology; C = absence of intervention, traditional interventions, or other approaches (when applicable in the included studies); and O = promotion of well-being, stress reduction, improvement in job satisfaction, or other indicators of mental health and quality of life in occupational settings. Based on this structure, the following guiding question was formulated: What empirical evidence is available on the effects of applying Positive Psychology principles or interventions to support the mental health and well-being of healthcare professionals?

### 2.3. Eligibility Criteria

Inclusion and exclusion criteria were established to ensure the relevance and theoretical-methodological coherence of the studies selected for this review. The inclusion criteria comprised empirical investigations—regardless of methodological design—that had healthcare professionals as their target population, explicitly applied Positive Psychology principles or interventions, and were conducted in healthcare work environments, such as hospitals, clinics, or primary care units. Additionally, only publications between 2001 and 2025, written in English, Spanish, or Portuguese, were considered. Studies were excluded if they focused exclusively on patients, even if conducted in healthcare contexts; if they were purely theoretical and lacked empirical data; if they addressed general well-being without explicit conceptual grounding in Positive Psychology; or if they described organizational or institutional interventions not conceptually based on Positive Psychology principles.

### 2.4. Search Strategies

The literature search was conducted across four major international databases with broad coverage in the fields of health sciences and psychology: Web of Science, Scopus, PubMed, and APA PsycArticles. The initial search strategy combined the descriptors “positive psychology” AND “healthcare professionals” OR “health workers” AND “intervention”. However, this formulation yielded an excessive number of irrelevant results, as evidenced in the Web of Science database, which returned 1963 records. Based on a preliminary review of titles and abstracts, it became clear that the search terms—especially the Boolean structure—needed refinement. The strategy was then reformulated as: “positive psychology” AND “healthcare professionals” AND “intervention”, with filters applied for document type (articles) and publication range (2021 to 2025). This time frame was justified by the focus on recent evidence, which aligns with the principles of scoping reviews.

Additionally, targeted searches were conducted in specialized journals in positive psychology, aiming to identify relevant publications that may not be indexed in the aforementioned databases. The journals consulted included the International Journal of Applied Positive Psychology, The Journal of Positive Psychology, Journal of Positive Psychology and Wellbeing, Revista Latinoamericana de Psicología Positiva, and International Journal of Positivity and Well-Being. In all journals, searches were performed using the isolated terms: “healthcare professionals” and “health workers”.

### 2.5. Screening and Selection of Evidence

**Figure 1:**
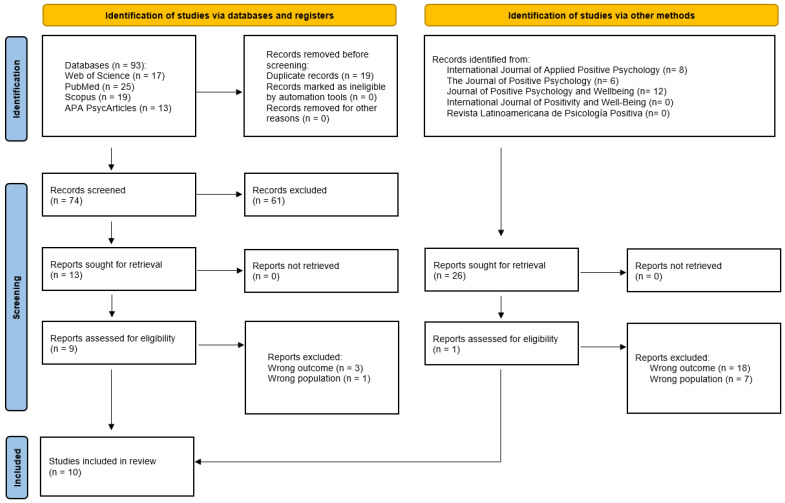
PRISMA 2020 flow diagram, which included searches of databases, registers and other sources.

All identified results were exported to the Rayyan platform [20]. The initial search across databases retrieved 93 records, while the complementary search yielded 26 articles. Duplicate entries were identified and removed. The screening process was conducted in two stages: first, a reading of titles and abstracts, followed by a full-text review of potentially eligible studies. The search flow and detailed outcomes are presented in Figure 1.

The main reasons for excluding articles were related to failure to meet the inclusion criteria, particularly regarding the target population (e.g., university students or patients instead of healthcare professionals) and outcomes not aligned with the guiding research question. Theoretical studies or those focused on outcomes unrelated to this review’s objectives were also excluded, especially during the full-text screening. Other exclusions involved studies with a distinct scope, focusing on exploratory investigations within the field of positive psychology or on unrelated approaches. In total, 10 articles were selected to comprise the corpus of this review [22,23,24,25,26,27,28,29,30,31].

### 2.6. Data Extraction

Data were extracted from the included articles using a synoptic chart developed exclusively by the lead researcher for this review. Extracted data included publication reference details, participants, contexts, methods, key results, and conclusions relevant to answering the guiding research question.

### 2.7. Data Analysis and Presentation

The data were analyzed descriptively and exploratorily. The findings from the reviewed studies are presented in tables, accompanied by a narrative summary that seeks to describe and establish connections between the results, the objective, and the guiding question of the review. The main findings were also examined using thematic analysis principles [32].

In this study, qualitative data analysis was supported by the use of the Requalify tool [33], a generative AI platform designed to assist in thematic coding and synthesis of qualitative research. The tool was employed specifically to enhance the organization and interpretation of thematic categories, without replacing the critical analytical role of the researchers. Its use adhered to current ethical guidelines for GenAI in research, ensuring transparency, accountability, and the preservation of human judgment throughout the interpretive process.

Additionally, a three-level analysis was conducted, titled META-CORE (Meta-level Conceptual, Operational, and Reflective Evaluation). This analytical model has been developed and refined by the author’s research group over the past five years. It was created in response to recurring challenges identified in reviews that do not follow traditional meta-analytic models, such as those commonly used in systematic reviews. It was observed, for instance, that many studies do not clearly distinguish between analytical processes beyond the descriptive level. This lack of definition or failure to report such processes compromises critical and comparative analysis of findings and does not produce a new synthesis of knowledge, which is a core function of literature reviews. Furthermore, the META-CORE guidelines were developed through the execution of several published literature reviews [34,35,36,37], as well as through systematic studies of methodological guidance on various types of review [38,39,40].

In this context, META-CORE proposes an analytical structure consisting of three levels: the conceptual (meta-theoretical) level, which examines the epistemological foundations and theoretical models guiding each study, and also considers bibliometric data such as authors, year of publication, and journal; the operational (meta-methodological) level, which analyzes the coherence between each study’s stated objectives, adopted methodological designs, and the way in which concepts are operationalized; and the reflective-evaluative (meta-synthetic) level, which assesses how results are synthesized, interpreted, and presented, including the articulation of limitations, inferences, and potential contributions to practice. At the meta-synthetic level, a new, evidence-based synthesis is also proposed. This analytical structure is presented in Table 1.

As previously mentioned, this analytical structure was developed within the research group coordinated by the lead researcher. Its inclusion in this article also constitutes an original contribution that may be applied to or examined by other researchers and scholars. Our experience in conducting literature reviews indicates that this analytical model, or way of approaching the data, is practical and effective, especially in contexts where meta-analyses or meta-syntheses are not being conducted. It is also important to highlight that at each level of analysis, a summary or descriptive synthesis should be presented to support a comprehensive and integrative view of the reviewed data.

## 3. Results

### 3.1. Meta-Level Conceptual

Ten articles were included in this review [22,23,24,25,26,27,28,29,30,31]. The majority of publications were concentrated in 2023 and 2024 (*n* = 7). The studies analyzed were conducted in diverse geographical contexts, reflecting different realities of healthcare professionals around the world. One study included ICU professionals from 55 countries across all global regions, offering a broad and comparative perspective on the well-being of these workers [22]. Specific studies were carried out in countries such as India [23,26], Spain [24,25], Malawi [27], Germany [28], Brazil [29], the United States [30], and Thailand [31].

The studies included in the review were published in well-established journals, primarily within the fields of health, psychology, and critical care. Among them, specialized journals such as Intensive and Critical Care Nursing and Intensive Care Medicine Experimental stood out, both focused on practices and evidence in intensive care. Multidisciplinary journals were also represented, such as BMC Health Services Research and the International Journal of Environmental Research and Public Health. In the field of psychology and well-being, studies appeared in journals like Behavioral Sciences, Current Psychology, and the International Journal of Applied Positive Psychology, as well as clinically oriented publications such as Contemporary Clinical Trials Communications and The Online Journal of Issues in Nursing. This variety reflects the diverse approaches through which positive psychology is being applied to support healthcare professionals, even within a relatively small body of reviewed studies.

An analysis of the authors’ professional profiles revealed the truly multidisciplinary nature of the reviewed topic, with contributions primarily emerging from the fields of psychology and health. In total, the ten articles reviewed involved approximately 79 unique authors, after removal of duplicate names. These authors held diverse and advanced qualifications, including doctoral degrees in fields such as nursing, psychology, public health, and methodology. Many of them also occupy prominent roles as professors, researchers, or clinical directors in healthcare institutions.

Table 2 presents the theoretical aspects highlighted in each article included in the review. These data relate to the identification of the theoretical, epistemological, and conceptual foundations of the reviewed articles, aiming to clarify the models that were mobilized and to what extent.

A strong theoretical anchoring in the core pillars of Positive Psychology was identified, with particular emphasis on three main strands: character strengths and personal virtues, positive emotions, and psychological well-being. Nearly all articles incorporated the character strengths model, as evidenced by the use of concepts such as resilience, gratitude, optimism, humor, and motivation [22,23,26,28]. In parallel, the focus on positive emotions [30,31] reflects an emphasis on affective development as a coping resource for occupational challenges and as a means of promoting well-being. At the same time, there is also a noticeable trend toward the practical application of Positive Psychology principles in caring for healthcare professionals [24,25,29]. For instance, one study offered a brief overview of Positive Psychology in general [25], while another conducted a literature review to justify or support the proposed research [26]. One study, in particular, presented a robust review of the literature on gratitude [30].

However, in some cases, theoretical foundations were either weak or lacked clear justification for the selection of constructs explored in the studies. Frequently, there was insufficient explanation as to why one or another aspect of Positive Psychology was chosen, nor was it clear how those variables were expected to connect to the intended outcomes. This issue already constitutes a meta-theoretical gap that future empirical or review studies may address.

### 3.2. Meta-Level Operational

The reviewed studies can be organized into two major groups based on their primary objectives. The first group comprises articles that aimed to evaluate interventions grounded in the principles of Positive Psychology within healthcare work settings. These studies prioritized practical approaches and were mostly designed as randomized or non-randomized clinical trials. The second group of studies focused on understanding positive psychological constructs and their correlates in occupational health contexts. This group also included one purely qualitative study. These data are presented in Table 3.

The methodological plurality identified allowed for the capture of both outcome measurement (quantitative studies) and the understanding of processes and meanings (qualitative studies). Similar and complementary patterns were also observed, revealing the robustness of the reviewed studies. Notably, several studies employed experimental or quasi-experimental designs focused on interventions, such as randomized clinical trials (e.g., ref. [22,29]), which enabled pre- and post-intervention comparisons and the monitoring of changes over time. Complementarily, other studies adopted cross-sectional or survey-based designs [25,31], using correlations and regression analyses to explore associations between positive variables, well-being, satisfaction, or performance.

It is also worth noting that most studies applied standardized and validated instruments, many of which originate from Positive Psychology or are related to occupational well-being (e.g., Maslach Burnout Inventory, the Flourishing Scale, or gratitude and resilience scales). This consistency supports the integration of results across different contexts. Furthermore, despite methodological differences, some studies converged toward a combined approach using both quantitative measures and qualitative or mixed-method analyses, reinforcing the multimethod nature and potential of research in Positive Psychology.

Although formal methodological quality assessment is not a requirement in scoping reviews [41], the present study opted to adapt key criteria from the AMSTAR 2 tool (A MeaSurement Tool to Assess Systematic Reviews) to ensure greater rigor in analyzing the included studies [42]. The adaptation focused on core elements of methodological clarity, such as the description of Population, Intervention, Comparator, and Outcome (PICO), as well as transparency in the presentation of data collection and analysis procedures. Based on these criteria, all ten reviewed articles were considered to have high methodological quality or non-critical weaknesses with low risk of bias, demonstrating solid research design and reporting.

### 3.3. Meta-Level Reflective Evaluation

This category includes the main findings reviewed. In this regard, the key results reveal a wide range of effects associated with the application or analysis of Positive Psychology principles in the care of healthcare professionals. Overall, the findings indicate benefits at both the individual and organizational levels. Although the methodologies and specific foci vary across studies, a consistent pattern of positive impacts can be observed, directly aligned with the theoretical pillars of interest in this review. A synthesis of these results is presented in Table 4.

**Figure 2:**
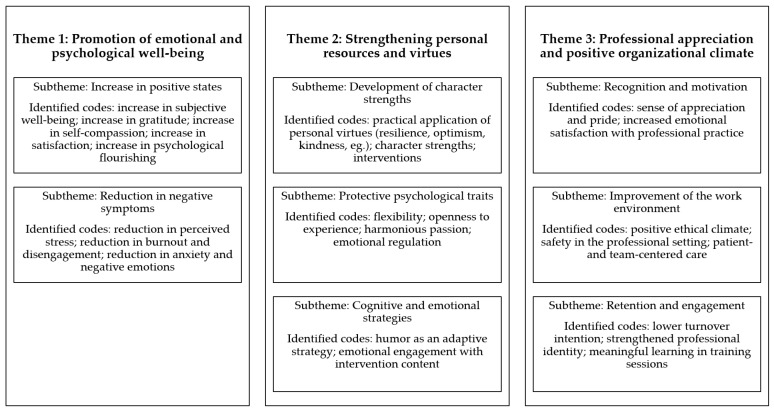
Coding tree of the main findings from the reviewed studies.

Based on the main findings from the reviewed studies, a coding process and thematic construction were conducted. In this process, the final coding tree consisted of 25 codes and eight subthemes, which were grouped into three overarching central themes. The complete coding tree is presented in Figure 2.

The first theme encompasses findings related to the reduction in stressors and the strengthening of healthcare professionals’ emotional states. Studies reported significant reductions in stress, burnout, anxiety, and negative emotions [23,29,30], along with increases in positive emotions such as gratitude and flourishing [30,31]. Additional evidence highlighted improvements in self-compassion and emotional regulation following contemplative-based interventions [24], and the use of humor as a positive resource to promote well-being and emotional resilience [28].

The second theme brings together studies that explored adaptive personal characteristics as protective factors for mental health. Interventions centered on character strengths were shown to be effective or potentially effective in promoting subjective well-being [23,26], and resilience and optimism emerged as core traits associated with positive outcomes [25,31]. Psychological flexibility and openness to experience were also identified as elements that support emotional adaptation [25], while engagement with practices grounded in virtues and positive emotions contributed to greater well-being and self-care [24].

Finally, the third theme concerns changes observed in the work environment and interpersonal relationships. Applications of Positive Psychology principles or related interventions that promoted professional recognition and the strengthening of workers’ identities led to increases in sense of purpose, pride, and collective well-being [22,27]. A positive impact on organizational climate was also observed, including higher perceptions of safety, ethical climate, and patient- and family-centered care [22]. In addition, humor was highlighted as a tool for strengthening team cohesion and promoting engagement [28].

### 3.4. New Synthesis and Implications for the Field of Health Promotion

The results allowed for the identification of new elements that reinforce and expand existing models of mental health promotion among healthcare professionals. Although not all studies demonstrated statistically significant effects in their quantitative outcomes, the qualitative data revealed meaningful changes in participants’ subjective experience, such as increased positive emotions, self-esteem, and sense of purpose. The analytical axes also provide a comprehensive overview of the dimensions that should be considered in care and prevention strategies. By articulating affective, cognitive, relational, and contextual elements, the reviewed studies support the relevance of interventions that go beyond symptom mitigation, aiming instead to foster healthier, more humane, and sustainable work environments. Thus, the evidence points to an expanded perspective on health promotion, aligned with the principles of Positive Psychology and collective health, in which the care of healthcare workers is understood as a strategic component for strengthening health systems.

## 4. Discussion

This study gathered up-to-date evidence on the effects of applying Positive Psychology principles or interventions in supporting the mental health and well-being of healthcare professionals. The three themes identified, which synthesize the main findings, demonstrate how the use of Positive Psychology principles can be beneficial in promoting emotional well-being, strengthening personal resources, and positively transforming the work environment. These principles contribute to reducing occupational stressors and to creating healthier, more motivating, and welcoming contexts. Moreover, the findings suggest that strengthening virtues such as gratitude, resilience, and optimism/humor may play a protective role against the negative impacts of intense and emotionally demanding work in healthcare, while also fostering greater engagement, retention, and sense of purpose in professional practice.

Given these results, an important area for further discussion concerns how to maintain the positive gains or the development of positive skills (such as increased subjective well-being, gratitude, or self-compassion) over time, and how to integrate them sustainably into daily routines amid mental distress and work overload in health professions. This points to a movement toward incorporating character strengths into professional contexts [15]. Thus, strengthening emotional and psychological well-being not only benefits professionals individually but also reinforce the role of healthcare workers as models of positive behavior in challenging settings [17]. This expands the notion of care to include not only technical competencies but also emotional and relational resources, grounded in self-awareness and emotional self-regulation.

Given this discussion, the question of how to sustain positive psychological skills over time becomes central, especially in demanding healthcare environments marked by emotional overload and chronic stress. Positive psychology, as the science of optimal functioning, offers conceptual tools to understand how these inner resources can be cultivated and maintained as part of everyday professional life. Rather than viewing well-being as a temporary outcome of isolated interventions, this perspective emphasizes its integration into the fabric of professional identity and organizational culture. Strengthening character strengths in this way not only enhances personal resilience but also transforms the quality of interpersonal relationships and teamwork, supporting healthcare professionals in becoming more emotionally attuned, reflective, and capable of self-regulation. This redefinition of care includes not only technical expertise but also emotional presence, empathy, and the ability to create spaces of psychological safety.

At the same time, the development of adaptive traits and character strengths—such as resilience, optimism, kindness, psychological flexibility, and openness to experience—appears to function as a protective factor against occupational suffering. These traits also have the potential to enhance engagement and professional satisfaction. For example, studies with healthcare professionals have shown that higher levels of resilience are associated with lower perceived stress, reduced fatigue, and greater job satisfaction, acting as a buffer against work-related demands [43]. Another study found that psychological flexibility mediated the relationship between resilience and academic burnout, suggesting that cultivating this trait could amplify the protective effect of resilience [44].

While many of these outcomes focus on the individual, it was also observed that the application of Positive Psychology principles has the potential to transform relationships and institutional practices, thereby promoting healthier work environments. Studies in organizational contexts show that Positive Psychology–oriented interventions, which promote the conscious use of such personal resources, not only enhance individual well-being but also lead to changes in relationships and practices within institutions, fostering more productive and supportive workplaces [45]. However, these findings should be interpreted with caution, and future studies should aim to evaluate organizational outcomes objectively and longitudinally, assessing the broader impact of changes informed by Positive Psychology.

The findings of this review still reveal a persistent underutilization of Positive Psychology as a proactive support framework for healthcare workers, despite growing evidence that interventions based on positive psychological principles can improve well-being and reduce burnout symptoms. Systematic reviews have shown that Positive Psychology Interventions (PPIs) delivered in workplace settings can lead to measurable improvements in psychological well-being, reductions in stress and burnout, and increases in resilience and job satisfaction among healthcare professionals [46,47]. Moreover, randomized studies indicate that online positive psychology programs can significantly decrease depression, anxiety, stress, and emotional exhaustion among clinicians, while enhancing life satisfaction [48]. These results suggest that public health strategies should shift from predominantly reactive stress-treatment interventions to integrated training models that build positive psychological resources before and during professional practice, enhancing both individual adaptability and systemic resilience.

It seems that practical measures to improve the effectiveness and humanization of care for healthcare professionals also involve the “depathologization” of strategies for coping with occupational stress. Rather than treating symptoms of distress only after they have manifested, the proposal is to redefine the focus: shifting from a model centered on correcting individual shortcomings to a preventive approach that acknowledges the emotional challenges of healthcare work as a structural reality. In this sense, this article argues that Positive Psychology can serve as a conceptual foundation to broaden the role of healthcare teams, integrating the promotion of well-being, resilience, and emotional self-regulation as essential components of everyday professional care.

All the reviewed studies also contribute to reflections on health promotion strategies for healthcare professionals, which must consider both the subjective and structural dimensions of work, recognizing professionals as active agents and legitimate recipients of care. For instance, a study involving newly graduated health and social care professionals found that supportive work conditions, autonomy, and positive internal experience (organizational structure) were strongly correlated with health-promoting resources (subjective dimension), suggesting that coordinated action between individuals and institutions is essential to promote sustainable well-being [49]. While this represents an initial discussion, it opens paths for further studies to explore health promotion and its guiding principles in alignment with Positive Psychology and its potential applications in interventions targeting healthcare professionals.

In terms of public policy impact, for example, the use of positive psychology to promote healthcare professionals’ well-being reveals potential not only for individual-level interventions but also for guiding structural decisions and institutional policies. The emphasis on personal resources, coping strategies, and a positive organizational climate, as identified in the scoping review, broadens the traditional scope of occupational health policies, which often focus on reactive responses to stress. This broader view aligns with the notion of positive sustainability, which proposes the integration of subjective well-being and positive values into long-term strategies for public policy planning. As argued by Tal and Kerret [50], a direct and proactive approach grounded in positive psychological principles can be applied to shape sustainable public policies. Similarly, in the field of occupational health, one can envision policies that not only prevent illness but also foster institutional environments that promote psychological flourishing, professional recognition, and the development of personal strengths. This perspective contributes to more integrated, sustainable policies that reinforce systemic care capacity.

## 5. Conclusions

This study synthesized evidence on the use of Positive Psychology in supporting healthcare professionals. It also offered reflections on the implications for promoting the mental health of professionals in the healthcare sector, particularly in light of the identified gap regarding the lack of intervention-based studies. In addition to the synthesized findings, a notable contribution of this study is the detailed presentation of the META-CORE model, which was applied to organize and analyze the reviewed data. The main findings underscore the importance of shifting from reactive stress treatment to proactive models of training that promote healthcare workers’ psychological well-being. Despite its demonstrated benefits, Positive Psychology remains underused in occupational health strategies. However, two main limitations of this review must be acknowledged. First, the methodological heterogeneity among the studies hinders direct comparison of findings and prevents the application of more complex analyses. Second, although all studies are aligned with the principles of Positive Psychology, many do not clearly describe the theoretical foundations underlying their interventions or methodological approaches, which limits understanding of how core concepts were operationalized. Further research is suggested to address or mitigate these limitations. At the same time, new intervention studies grounded in Positive Psychology are recommended to promote the mental health or professional support of healthcare professionals.

## Figures and Tables

**Table 1:** Analytical structure of the META-CORE Model.

Analytical Level	Description	Guiding Questions	Data to Be Collected
Conceptual (Meta-theoretical)	Assesses the theoretical, epistemological, and conceptual foundations of the reviewed articles. It seeks to understand which models are mobilized and to what extent. It also considers the context of scientific production, identifying patterns related to authorship, journals, countries, and disciplinary areas.	What theoretical framework is stated? What key concepts are used? How do the authors justify the use of these concepts or their theoretical choices? Who are the authors? What are their institutional affiliations or fields of expertise? In which journals were the articles published? When? In which countries were the studies conducted?	Citations of authors or founders of theories. Adopted conceptual definitions. Referenced theoretical models. Level of theoretical grounding (explicit, implicit, absent). Journal title, year of publication, country of origin of the research. Type of journal (psychology, health, interdisciplinary, etc.).
Operational (Meta-methodological)	Analyzes the coherence between the conceptual assumptions and the methods employed, including study design, participants, adopted measures/instruments, and context. Includes assessment of methodological quality when necessary or recommended.	What are the main characteristics of the study’s method? How was the study designed and conducted? What instruments and indicators were used? Is the context (setting, population) well described?	Type of design. Procedures. Measures/instruments used. Description of the context and target population. Type of analysis performed. Use of tools.
Reflective-Evaluative (Meta-synthetic)	Examines how the results are interpreted, integrated, and discussed, including study limitations, articulation with the literature, and practical implications. Focuses on the creation of a new synthesis—a new knowledge product.	Are the results discussed in light of the theoretical framework? What are the main findings presented? Are the study’s limitations acknowledged? Are there recommendations for practice and future research? Does the study contribute to consolidating, questioning, or expanding the field?	Synthesis strategy (e.g., categories, themes, metrics). Discussion of limitations. Connection between results and theory. Authors’ conclusions and recommendations. Production of a new synthesis integrating the main findings from all reviewed studies.

**Table 2:** Verification of the use of positive psychology and declared theoretical foundations.

Reference	Statement of Positive Psychology Use	Highlighted Theoretical Aspects
[22]	yes	Positive emotions (happiness, joy, and contentment) Individual strengths and virtues, such as resilience, gratitude, and optimism
[23]	yes	Character strengths Psychological well-being
[24]	yes	Concept of contemplative positive psychology, understood as a domain that includes techniques and conceptual frameworks developed by contemplative sciences to promote well-being through evidence-based strategies Virtuous actions, emotional regulation, compassion, character strengths, appreciation, kindness, or the “three good things”
[25]	yes	Personal strengths Definition of positive psychology is a field that investigates the emotions, strengths, processes, conditions, and relationships that promote optimal functioning and well-being in individuals, groups, and institutions Optimism, defined as positive expectations regarding the outcomes of future events Resilience is one of the positive personality traits
[26]	yes	Character strengths Values in Action = personal value systems, referred to as “virtues,” and their underlying strengths Positive personality traits
[27]	yes	Personal strengths Motivation
[28]	yes	Humor as a character strength Humor styles Sensitivity to assess whether humor is appropriate (e.g., empathy, emotion recognition)
[29]	yes	Happiness and human potential More optimistic aspects of personality Development of virtues, quality of life, and well-being Positive psychology interventions
[30]	yes	General well-being and life satisfaction Personal strengths Resilience Gratitude, as an affective trait defined as a cognitive process Positive emotions
[31]	yes	Physical and psychological well-being Compassion satisfaction, referring to the happiness or fulfillment people experience when helping or caring for others who are suffering Cognitive skills, such as problem-solving and effective decision-making Positive psychological constructs, including resilience, passion, psychological flexibility, and flourishing

**Table 3:** Overview of the objectives and methodological data of the reviewed articles.

Reference	Objectives	Methodological Data
[22]	Evaluate the impact of an intervention on the prevalence of burnout among healthcare professionals working in intensive care units (ICUs); compare burnout rates before and after the intervention	Randomized clinical trial [proposed] 434 ICUs were randomized (217 in the intervention group and 217 in the control group) Instruments used: informational emails and slides; instructional video; Maslach Burnout Inventory (MBI) Statistical analyses: descriptive statistics; bivariate comparisons; multivariate modeling Software used: R statistical software, version 3.4.3
[23]	Test the effectiveness of a character strengths-based coaching intervention compared to routine supervision in enhancing well-being (“authentic happiness”)	Randomized clinical trial [proposed] 330 community health workers Instruments used: Authentic Happiness Inventory; Positive and Negative Affect Schedule (PANAS) Short Form; Flourish Index; Occupational Self-Efficacy Scale (OSES); Maslach Burnout Inventory—Human Services Survey; Motivation Scale pretested for Indian frontline health workers; 14-item Physical Health Questionnaire; EQ-5D Health-Related Quality of Life Survey Statistical analyses: descriptive statistics; correlations; regression models; difference in proportions test Software used: statistical software not explicitly mentioned
[24]	Investigate the effectiveness of a contemplative practice-based well-being training program for enhancing psychological functioning	Non-randomized clinical trial Sample: 38 professionals from ICUs and home care services (19 in the intervention group and 19 in the control group) Instruments used: Modified Differential Emotions Scale (mDES); Self-Compassion Scale—Short Form (SCS-SF); Difficulties in Emotion Regulation Scale-18 (DERS-18); Maslach Burnout Inventory (MBI) Statistical analyses: descriptive statistics; mixed MANOVA Qualitative analysis: thematic analysis Software used: statistical software not explicitly mentioned
[25]	Analyze the influence of openness to experience and resilience on the development of optimism	Cross-sectional study Sample: 151 nurses Instruments used: sociodemographic questionnaires; concerns about self-contagion; Spanish version of the Big Five Inventory-10 (BFI-10); Occupational Hardiness Questionnaire (OHQ); Spanish version of the Life Orientation Test-Revised (LOT-R) Statistical analyses: descriptive statistics; correlations; regression models; ANOVA Software used: PROCESS macro (Model 7)
[26]	Describe a systematic approach to planning and developing a character strengths-based coaching program for community health workers	Methodological study Sample: [from two study phases] psychologists with experience in community mental health (*n* = 3), positive psychology experts (*n* = 3), government officials with extensive experience training community health workers (*n* = 2), and senior researchers (*n* = 4); 15 community health workers Procedures: formative groups; literature review; development of the intervention program plan; development of intervention content; pilot study with community health workers Qualitative analysis: thematic analysis
[27]	Evaluate the feasibility, outcomes, and impact of a program called “Learning from Excellence”	Mixed-methods study Sample: 390 community health workers in the quantitative phase; in the qualitative phase, 14 community health workers, 7 local supervisors, and 3 community leaders Instruments used: researcher-developed questionnaire; interview script Statistical analyses: descriptive statistics; correlations; regression models Qualitative analysis: thematic analysis Software used: statistical software not explicitly mentioned; qualitative data processed using NVivo
[28]	Investigate the potential of humor as a resource for work and professional education	Qualitative study Sample: 14 attending physicians Procedure: individual interviews using a standardized interview guide Data analysis: qualitative content analysis with a combination of inductive and deductive coding Software used: MAXQDA
[29]	Evaluate the effects of a multicomponent intervention based on meditation and Positive Psychology in reducing stress	Randomized clinical trial Sample: 29 female healthcare professionals (intervention = 14; control = 15) Procedures: Flourish Program = multicomponent intervention consisting of 90 min weekly sessions over eight weeks, accompanied by daily meditation practices Instruments used: Perceived Stress Scale (PSS); Self-Report Questionnaire-20 (SRQ-20); Beck Depression Inventory (BDI); Beck Anxiety Inventory (BAI); Pittsburgh Sleep Quality Index (PSQI) Statistical analyses: descriptive statistics; correlations; regression models; ANOVA Software used: JASP program (Version 0.13)
[30]	Evaluate the effect of participation in a 21-day therapeutic writing intervention on levels of gratitude	Prospective pre/post study with follow-up Sample: 405 healthcare professionals (initial group); 45 healthcare professionals (completers group) Instruments used: Gratitude Questionnaire—Six Item Form (GQ-6); Perceived Stress Scale—4 Item; Oldenburg Burnout Inventory; Demographic and Clinical Characteristics Statistical analyses: descriptive statistics; correlations; regression models Software used: SPSS 25 software (version 23.0)
[31]	Determine the prevalence of compassion satisfaction, identify associated factors, and determine predictors of compassion satisfaction	Cross-sectional study Sample: 164 nurses; 14 physicians Instruments used: Professional Quality of Life Scale Version 5; Connor-Davidson Resilience Scale; Passion Scale; Flourishing Scale; Acceptance and Action Questionnaire Statistical analyses: descriptive statistics; correlations; regression models Software used: SPSS 28 software (version 28.0)

**Table 4:** Main findings from the reviewed studies.

Reference	Key Findings Related to the Review Objective and Guiding Question
[22]	The intervention is expected to reinforce positive interactions, recognition, and positive communication. The intervention group is expected to show higher job satisfaction, better ethical climate, greater perceived safety in the work environment, more patient- and family-centered care, and lower intent to leave ICU work.
[23]	The character strengths-based intervention is expected to lead to a significant increase in subjective well-being among community health workers in the intervention group. Reduction in occupational stress and burnout.
[24]	Healthcare professionals who participated in the intervention reported a significant decrease in negative emotions. Evidence suggests that the intervention contributed to improvements in emotional regulation. Participation in the activities may have served as a protective factor against loss of professional purpose or efficacy. Participants in the intervention reported increased levels of self-compassion after completing the program. Six themes were extracted from the qualitative analysis: improved emotional regulation, greater awareness, enhanced well-being, increased appreciation, better self-care and care for others, and knowledge acquisition.
[25]	Professionals with greater openness to experience tended to develop higher resilience over time, which in turn was associated with higher levels of optimism. Psychological flexibility associated with openness may function as a protective factor, facilitating the development of emotional resilience. Significant correlations were observed between resilience and optimism, and between resilience and openness to experience.
[26]	The pilot study activities were effective in generating active engagement and emotionally meaningful experiences for participants. Reports of satisfaction, enthusiasm, and emotional lightness after the sessions described the training as different and more welcoming than typical technical programs. The methods used (e.g., role-playing, videos, games, and practical activities) were recognized as facilitators of learning and personal identification with the topics.
[27]	The intervention made community health workers feel valued and recognized. Many reported emotional satisfactions, joy in recognizing peers, and well-being in being acknowledged. Increased sense of professional pride, strengthening of purpose, and professional identity. The program enabled the identification and replication of good practices.
[28]	The use of humor was widely recognized as an essential strategy for promoting emotional well-being, increasing motivation at work, and improving both individual and team performance. Humor contributed to the strengthening of interpersonal relationships and team cohesion, and facilitated empathetic connection with patients. The use of humor enabled resilient coping with stressful situations, mistakes, and limitations. Humor served as an educational and inspirational tool with trainees, promoting self-confidence, engagement, and the internalization of positive attitudes.
[29]	Although no statistically significant differences were observed in depression, psychiatric symptoms, or sleep quality between groups, positive trends were noted, especially in the intervention group. The intervention showed significant effectiveness in reducing perceived stress and anxiety symptoms after 8 weeks, particularly in the intervention group.
[30]	Gratitude significantly increased after the intervention and remained elevated at 3-month follow-up, with a small to medium effect size, suggesting a lasting positive impact. Higher levels of gratitude were associated with lower levels of perceived stress, disengagement, and emotional exhaustion. A protective relationship between gratitude and psychological well-being was confirmed.
[31]	Compassion satisfaction showed strong positive correlations with resilience, flourishing, and harmonious passion. Regression analysis identified resilience as the strongest predictor of compassion satisfaction, followed by harmonious passion. Preliminary, non-significant data suggested that greater psychological rigidity may be linked to lower satisfaction, while professional maturity and experience may contribute to increased well-being.

## Data Availability

All data supporting the findings of this study are available within the published article. Interested researchers may contact the corresponding author for further information or clarification regarding the reviewed materials and analytical procedures.

## Supplementary Materials

The following supporting information can be downloaded at: https://www.mdpi.com/article/10.3390/ijerph23030296/s1, File S1: PRISMA checklist.
